# Network analyses to quantify effects of host movement in multilevel disease transmission models using foot and mouth disease in Cameroon as a case study

**DOI:** 10.1371/journal.pcbi.1007184

**Published:** 2019-08-29

**Authors:** Laura W. Pomeroy, Hyeyoung Kim, Ningchuan Xiao, Mark Moritz, Rebecca Garabed

**Affiliations:** 1 Division of Environmental Health Sciences, College of Public Health, The Ohio State University, Columbus, OH, United States of America; 2 Department of Geography, The Ohio State University, Columbus, OH, United States of America; 3 Department of Disease Control and Epidemiology, National Verterinary Institute, Uppsala, Sweden; 4 Department of Anthropology, The Ohio State University, Columbus, OH, United States of America; 5 Department of Veterinary Preventive Medicine, The Ohio State University, Columbus, OH, United States of America; University of California Irvine, UNITED STATES

## Abstract

The dynamics of infectious diseases are greatly influenced by the movement of both susceptible and infected hosts. To accurately represent disease dynamics among a mobile host population, detailed movement models have been coupled with disease transmission models. However, a number of different host movement models have been proposed, each with their own set of assumptions and results that differ from the other models. Here, we compare two movement models coupled to the same disease transmission model using network analyses. This application of network analysis allows us to evaluate the fit and accuracy of the movement model in a multilevel modeling framework with more detail than established statistical modeling fitting methods. We used data that detailed mobile pastoralists’ movements as input for 100 stochastic simulations of a Spatio-Temporal Movement (STM) model and 100 stochastic simulations of an Individual Movement Model (IMM). Both models represent dynamic movement and subsequent contacts. We generated networks in which nodes represent camps and edges represent the distance between camps. We simulated pathogen transmission over these networks and tested five network metrics–strength, betweenness centrality, three-step reach, density, and transitivity–to determine which could predict disease simulation outcomes and thereby be used to correlate model simulation results with disease transmission simulations. We found that strength, network density, and three-step reach of movement model results correlated with the final epidemic size of outbreak simulations. Betweenness centrality only weakly correlated for the IMM model. Transitivity only weakly correlated for the STM model and time-varying IMM model metrics. We conclude that movement models coupled with disease transmission models can affect disease transmission results and should be carefully considered and vetted when modeling pathogen spread in mobile host populations. Strength, network density, and three-step reach can be used to evaluate movement models before disease simulations to predict final outbreak sizes. These findings can contribute to the analysis of multilevel models across systems.

## Author summary

Epidemics of infectious disease vary geographically and vary through time. A large part of this variation is caused by movement of individuals who are susceptible to the disease or infected with the disease. To study how movement affects epidemics, researchers often combine movement models with transmission models. However, multiple movement models have been proposed, and their effect on infectious disease model output is not well understood. Here, we combine two different movement models that we developed to represent mobile pastoralists in the Far North Region, Cameroon, with the same disease transmission model. We use network metrics to test how different movement models can affect the output of the disease transmission model. We found that three metrics could be applied to movement model output in order to predict epidemic model output. We conclude that movement models coupled with disease transmission models can affect disease transmission results and should be carefully considered and vetted when modeling epidemics.

## Introduction

Host movement influences disease dynamics [1–3]. In situations where the population size is too small to sustain long-term chains of transmission, movement of infected hosts can spark local outbreaks and increase incidence counts. Alternatively, migration of susceptible hosts away from outbreak locations can reduce abundance or local density of the susceptible population, reduce contact rates, and decrease incidence counts or outbreak duration [1]. In order to quantify and predict pathogen transmission in a mobile host population, researchers couple mathematical and statistical models of disease dynamics with host movement models to more accurately represent local abundance of diseased hosts in a multilevel modeling framework [4–9]. The problem still remains, however, of how to best represent host movement since host behavior is complex and full movement trajectories are rarely observed.

To account for heterogeneity in host movement, multiple models have been proposed. These models range from stochastic models of movement, such as the set of random walk models [10], to more mechanistic models of movement [11], including distance-based and gravity-based movement models [12, 13]. Each movement model has its own set of assumptions and simulated movement trajectories can differ. When different movement models are coupled with disease transmission models [4–9] the movement model selected can impact results and conclusions relating to disease dynamics. It is important that the movement model used in a multilevel modeling framework with a disease transmission model accurately represent contacts that result in disease transmission. Statistical methods have been developed to resolve this specific problem and help find the most parsimonious movement model (reviewed in [14] and [15]).

One approach is to develop multilevel models that incorporate alternative hypothesized modes of movement and use statistical techniques to find which model fits the data best. For example, one multilevel model would include a movement model at level one and a disease transmission model at level two; another multilevel model would include a different movement model at level one and the same disease transmission model at level two. Using classical methods, one could find which multilevel model best fits the data using a goodness of fit method–e.g., maximum likelihood–and infer which movement model best represents movement patterns in the host population [15]. This approach works well when host location and disease incidence data are detailed enough to accurately select the most parsimonious model and when the outcome desired is the selection of the best-fit model at the population level. However, when model fit varies through time or by host, additional methods are needed for model evaluation and selection.

Here, we extend traditional methods of model selection by developing a suite of network analysis tools for multilevel disease transmission models that incorporate host movement. Networks consist of nodes, which represent hosts, that are connected by edges, or contacts among nodes through which pathogens propagate. This network approach has three advantages. First, networks permit flexibility in representing heterogeneous behavior that can affect disease transmission and overcome homogeneous behavior assumed in some traditional models [16]. Second, networks permit flexibility in representing host-pathogen systems–as the edge can represent any relevant mode of transmission–and surmount the need to develop specific methodology for each pathogen transmission scenario [17–19]. Third, networks can be implemented in a way that displays and measures effects of the movement level in a multilevel model even if these effects vary through time or vary by host. Together, these advantages highlight the usefulness of network analysis for model evaluation and selection.

To demonstrate our network analysis tools, we use livestock data from the Far North Region, Cameroon. This Region hosts many important livestock diseases–including five different serotypes of foot-and-mouth disease virus [20, 21]–making disease quantification and prediction important here. Host movement shapes disease transmission, because 7% of the estimated 650,000 cattle in this area participate in seasonal transhumance as a livestock management practice [22]. During transhumance, herders move their camps, which consist of mixed-species livestock herds and households, over great distances (>100 km). While pastoralists follow the same general patterns of movement [23], detailed spatiotemporal characteristics vary between herds. Each pastoralist makes a decision about the location of the campsite and the dates of arrival and departure and considers multiple factors for each move [24], suggesting that movement is a complex process.

To account for the uncertainty regarding in pastoralist movements in the Far North Region, Cameroon, we developed two movement models that represent camp movement and resulting contacts as dynamic processes. The first model is a spatial-temporal mobility model (STM) that estimates the probability of pastoralist movements based on detailed movement survey data [25]. This model categorizes pastoralists into three groups based on the shape of their movement trajectories and assigns corresponding probability distributions to herd locations [26]. The second model is called Individual Movement Model (IMM) and uses a set of rules in an agent-based simulation of pastoralist movements. This model assigns a herd to a sequence of seasonal grazing areas with random movements within these seasonal grazing areas [27]. We couple these movement models with disease transmission models to develop a multilevel modeling framework to quantify incidence among livestock in the Far North Region.

In this work, our objective is to determine how well each network analysis tool quantifies the effect of movement model selection and its impact on disease simulations, considering both temporal variation and the effect of host variation. First, we generate networks from stochastic simulations of both cattle movement models and analyze these networks. Second, we simulate and quantify disease transmission across networks stochastically generated from both cattle movement models. Finally, we determine which network connectivity metrics correlate with simulated disease incidence. By evaluating the accuracy of competing movement models and quantifying the impact of movement models on predicted outbreaks in this system with consideration of temporal variability and the effect of herd characteristics, we contribute information that could help control of infectious diseases among these mobile pastoralists. More broadly, we contribute a set of model evaluation tools that can be applied across systems quantified and analyzed with multilevel models.

## Materials and methods

### Location data

Location data were obtained from 72 mobile camps, which comprise a near complete set of all Cameroonian mobile pastoralists who visited the Logone Floodplain on any date between August 16, 2007 and August 15, 2008 [25]. Each camp contained, on average, approximately 8 households, 30 people, and 600 cattle [24]. Data were collected in two phases. First, locations of pastoral households were observed two times–in February 2008 and August 2008 –and the global positioning system (GPS) coordinates of campsite locations were recorded. Second, locations of pastoral households were obtained by structured interviews in August and September of 2008. These interview data represent campsite location at a finer temporal scale and inserted information about campsite location at dates that fell between first phase data collection events, thereby documenting the entire annual transhumance. Herders provided names of all locations in which they erected a campsite during the previous year and number of days spent at each location. Members of the research team visited each of the places that pastoralists listed in the transhumance interviews to obtain geographic coordinates. The full location data set consists of the GPS coordinates of the centroid of each named location for every date between August 16, 2007 and August 15, 2008. The data set is publically available at MoveBank [28].

### Ethics statement

The data collection protocol was reviewed and approved by the Ohio State University Institutional Review Board / Human Research Protection Program (Federal-wide Assurance #00006378 from the Office for Human Research Protections in the Department of Health and Human Services: protocol 2010B0004). We obtained informed consent after explaining the protocol and potential risks.

### Generating networks

**Fig 1:**
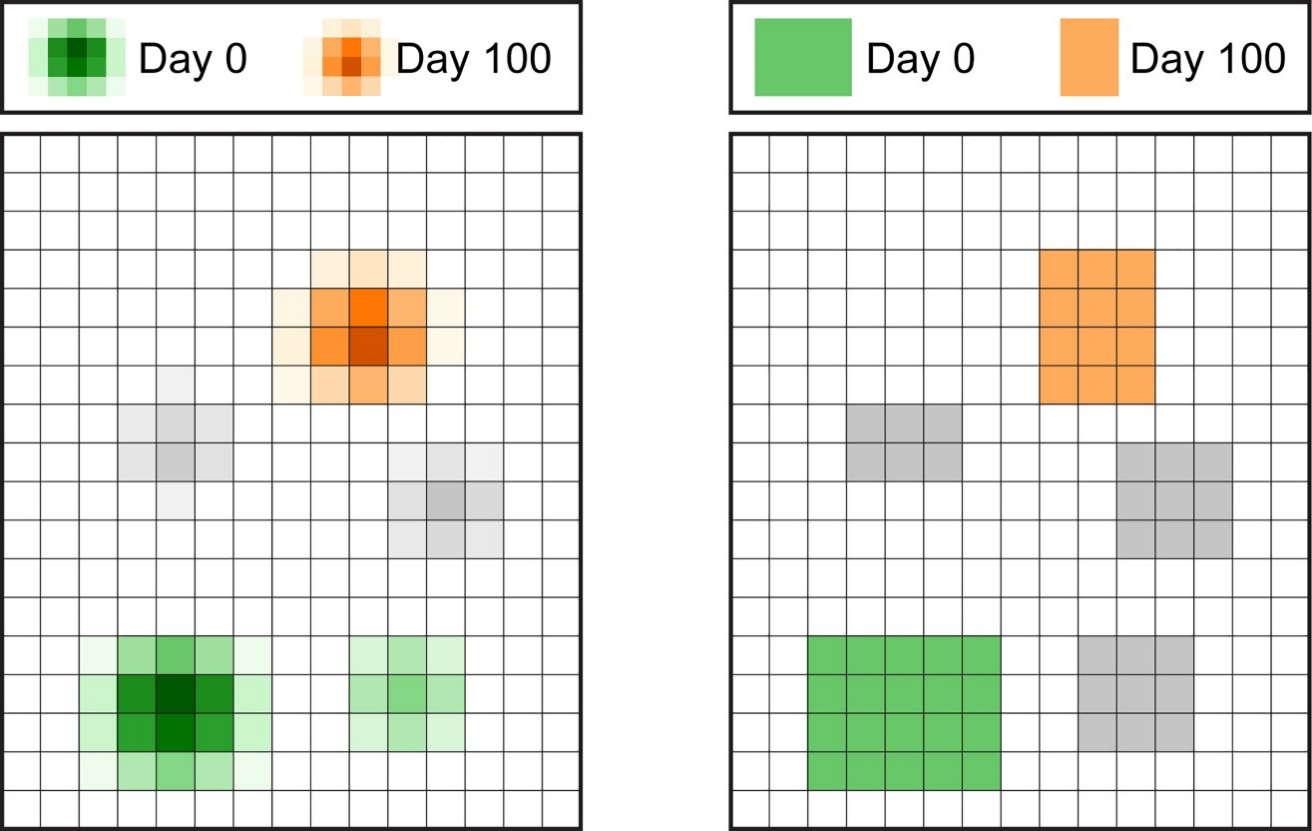
Differing methods for selecting locations in the STM and IMM models. In this simplified example, consider five locations among which one particular mobile herd moves. The probability of the herd being assigned different locations on Day 0 of the simulation is designated in green with the intensity of the color representing the probability, i.e. dark green = higher probability, light green = lower probability, no green = no probability. Similarly, the probabilities of this herd being assigned to different locations on Day 100 are designated in orange. Areas used by the herd at other times of the year are designated in gray. Left panel: The STM model generates a novel probability distribution for the herd’s location each day. Right panel: The IMM model has the herd moving through a sequence of zones with stochasticity in timing of movements and equal probability for being assigned to all locations within a target zone.

Location data are input for stochastic simulations of the STM and IMM models, both of which model camp movement and resulting contacts dynamically. The STM model simulates 71 mobile camps and the IMM model simulates 67 mobile camps (Fig 1). We ran 100 simulations of the STM model and 100 simulations of the IMM model [26, 27]. These produced 200 sets of simulated locations from which we generated networks.

To generate the networks, we first calculated symmetrical daily adjacency matrices in which we defined adjacency as all camps with simulated locations within a given distance *k*. If two camps, *i* and *j*, were separated by *k* km or a shorter distance, the {*i*,*j*} entry of the daily adjacency matrix would contain a one; if two camps, *i* and *j* were separated by more than *k* km, the {*i*,*j*} entry of the daily adjacency matrix would contain a zero.

We defined adjacency by setting *k* at three different distances: 0 km, 5 km, and 10km. At 0 km, camps are have simulated locations at the same coordinates, and share the same simulated location by definition. In practice, a single coordinate was chosen to represent many camps located at the same general campsite. At 5 km, we consider camps that have overlapping cattle grazing zones to be connected, because the grazing radius of a camp in the Far North Region is approximately 5 km [29]. At 10 km, we consider camps that have adjacent cattle grazing zones to be connected, again based on the 5 km grazing radius. Daily adjacency matrices were calculated for each of the 366 days for which location data and model outputs were available, because 2008 was a leap year. In total, we calculated 3 daily adjacency matrices for each of the 200 sets of simulation locations generated from two movement models, which resulted in a total of 219,600 daily adjacency matrices for the 2007–2008 year.

### Disease simulation across networks

To simulate disease across networks, it is important that the adjacency matrices summarize a duration equal to the infectious period of the pathogen [30]. Since the infectious period of foot-and-mouth disease is approximately one week in cattle [31], we summarized the daily adjacency matrices as weighted weekly adjacency matrices. To calculate the weighted weekly adjacency matrices, we summed daily matrices over seven days, resulting in 31,200 matrices. From these weekly matrices, we generated 31,200 weighted weekly networks.

We simulated disease transmission across weekly networks using a susceptible, infected, recovered (SIR) framework in which each herd was categorized in a single disease state and transmission occurred between herds. Susceptible herds had not previously experienced disease, were immunologically naïve, and capable of catching the pathogen. Infected herds harbored the pathogen and were capable of transmitting the pathogen to susceptible herds. Recovered herds mounted convalescent immunity after experiencing infection and were removed from chains of transmission [32].

Transmission propagated through the networks when an infected herd was connected to a susceptible herd by an edge. We simulated various values of *R*_0_, or the number of secondary cases produced by a primary infectious case in a completely susceptible population. We performed transmission simulations under three values for *R*_0_ (1, 5, 10), looping through the following protocol until no additional nodes could be infected.

1. For each infectious node *j*, *l* susceptible adjacent nodes were identified. Let *N*_*j*,*l*_ represent the number of susceptible adjacent nodes.
2. If the number of susceptible adjacent nodes was less than or equal to *R*_0_, such that *N*_*j*,*l*_≤*R*_0_, then all susceptible adjacent nodes *l* were infected.
  $$I_{t+1,k}=N_{j,l}$$ (1)
3. If the number of susceptible adjacent nodes was greater than or equal to the assigned number of secondary cases, such that *N*_*j*,*l*_≥*R*_0_, then *R*_0_ susceptible adjacent nodes were randomly chosen using the *sample* function in R and infected.
  $$I_{t+1,l}=\left(\begin{array}{c} N_{j,l}\\ R_{0} \end{array}\right)$$ (2)
4. Camps moved to the recovered state after one week, following foot-and-mouth disease pathology [31], and remained in the recovered state for the rest of the simulation.

This transmission propagation procedure keeps the number of secondary infections caused by each infectious node relatively constant and not greater than the value at which *R*_0_ is set. The nodes receiving the secondary infection are randomly chosen from the set of all possible nodes connected to the infectious node. We assume that cattle herds are infectious for 7 days, which is longer than the mean duration of infectiousness of an individual animal (4.5 days [33]), but implies that most of the herd gets infected within days. This duration was chosen considering the size of herds in the Far North Region, Cameroon, the proximity of animals in these mobile herds, and our assumption that the entire herd is susceptible.

We performed multiple disease transmission simulations for each value of *R*_0_ for a maximum of 366 days. Disease was initiated at each node and on each day, with simulations lasting from the day of initiation to the end of the 366-day period. For each of the 31,200 weighted weekly adjacency matrices, we performed simulations at 3 different *R*_0_ values, with disease initiated on each of the 52 weeks, for a total of 4,867,200 disease transmission simulations.

### Network analyses for model comparison

We used five different connectivity metrics to compare the structure of weekly networks: three node-level metrics averaged across the entire network and two network-level metrics [34]. The node-level metrics were strength, betweenness centrality, and 3-step reach. Strength is calculated for each node by summing the weights of all connected edges. Strength is a single metric that represents both the number of connections between the given node and all other nodes plus the duration of those connections. Betweenness centrality is calculated for each node by counting the number of shortest paths between any two other nodes on which the node lies; to do this, we used the *betweenness* command in R. This metric represents how many other nodes a given node plays a role in connecting; in terms of disease transmission, a camp with high degree centrality is crucial for propagating disease transmission. Three-step reach is calculated by counting the number of nodes within three edge lengths or three “steps” from any given node. This metric indicates how connected the entire network is; if three-step reach is high, an epidemic will propagate though a large part of the network in three time steps. The network-level metrics were network density and network transitivity. Network density is calculated as the sum of weighted adjacency matrices divided by the total number of edges possible in the network. This metric is a different way to calculate how connected the entire network is and also has implications for the speed and extent of infectious disease transmission. Network transitivity is calculated as a ratio where the numerator is the proportion of closed triads and the denominator is the proportion of open triads [34]. This metric measures the cliquishness of a network; infections initiated in a network with high transitivity will show pockets where disease transmitted and other pockets that the disease would not reach. To measure transitivity, we used the *transitivity* command in R.

To compare the two movement models, we calculated the value for each of these 5 network metrics for each of the 31,200 weekly adjacency matrices. We quantified how the 5 metrics correlated with the final epidemic sizes of disease simulations using Pearson’s correlation coefficient and p-value implemented with the *cor*.*test* function in R. The metrics that correlated well with outbreak size can be used as tools for analysis of the movement level of a multilevel model.

Because network characteristics might not immediately correlate with infectious disease spread, we quantified temporal variation in the movement model network metrics and disease simulation results. We tested lag periods of 0 weeks, 1 week, 2 weeks, 3 weeks, 4 weeks, and 5 weeks. For each lag period, we used Pearson’s correlation coefficient described to quantify the correlation between connectivity metrics and outbreak size. For each connectivity metric, we identify maximum correlation coefficient and report it, along with the time period that produced the maximum correlation coefficient. This method detects correlations that might have been obscured because they did not happen simulatelously with an increase in final epidemic size.

## Results

Here, we report results from analyses conducted when adjacency was defined at 5 km. Results from analyses conducted when adjacency was defined at 0 km and 10 km are reported in the supporting information.

**Fig 2:**
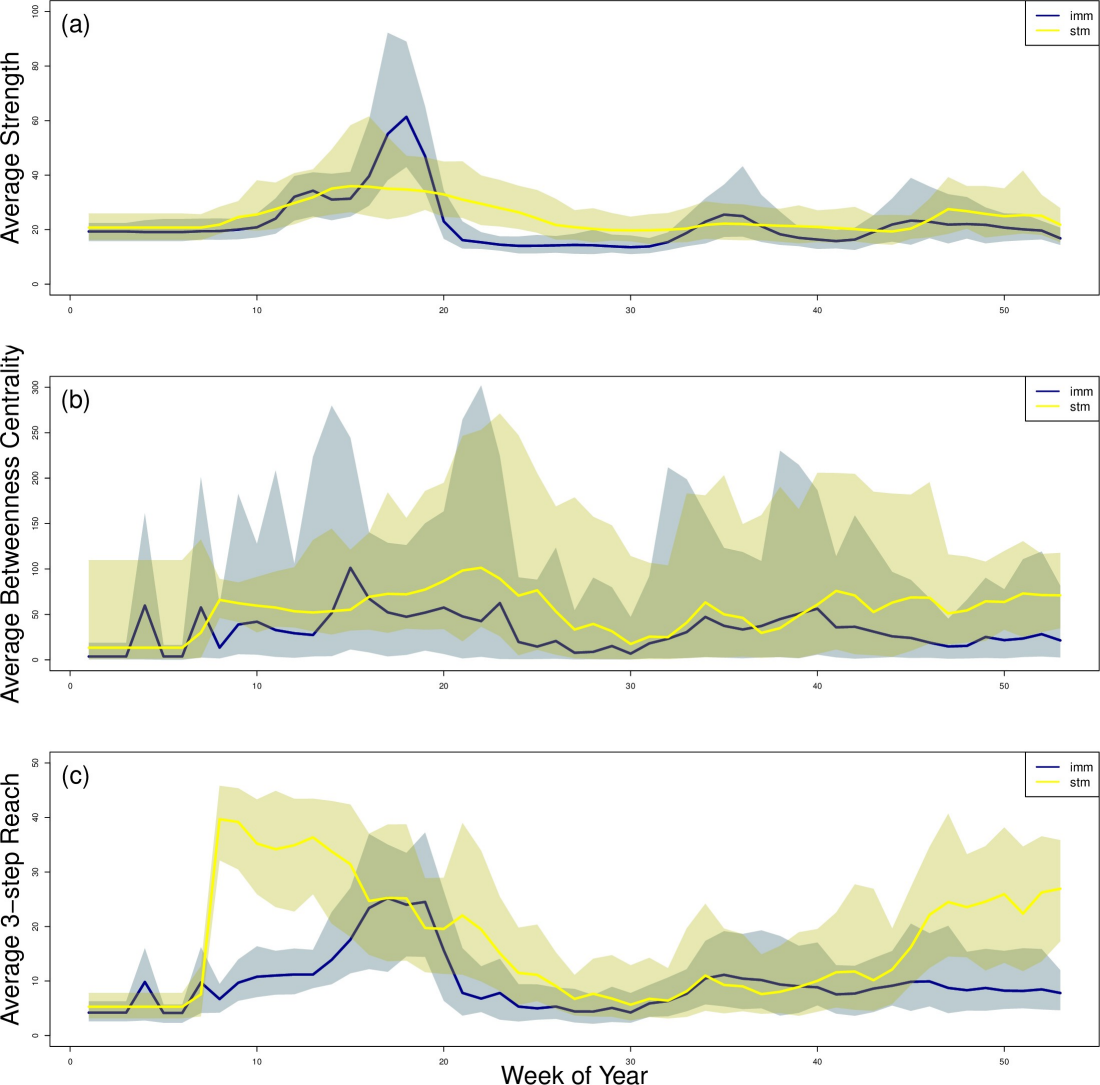
Node-level network analysis metrics of IMM and STM simulations with adjacency defined at 5 km. For 100 stochastic simulations of the IMM and 100 stochastic simulations of the STM, we calculated (a) average strength, (b) average betweenness centrality, and (c) average 3-step reach. We averaged these values over all nodes in a single network. The dark line represents the average values across all simulations. The shaded areas represent the range between the largest mean value and smallest mean value across all simulations. The metrics varied through time and varied by movement model simulated.

In order to quantify network connectivity with node-level metrics, we found the strength, betweenness centrality, and 3-step reach for each node in the weekly networks for 100 simulations of the IMM and 100 simulations of the STM and averaged these values across each network (Fig 2). Average strength in the IMM and STM were similar, especially at the start and end of the year; however, average strength showed three peaks in the IMM simulations that were not observed in the STM simulations (Fig 2A). Average betweenness centrality showed small peaks in the IMM and STM simulations that differed in timing (Fig 2B). Average 3-step reach was greater in the STM simulations than in the IMM simulations and showed greater peaks in the STM simulations (Fig 2C). In this way, the connectivity differed between the two movement models.

**Fig 3:**
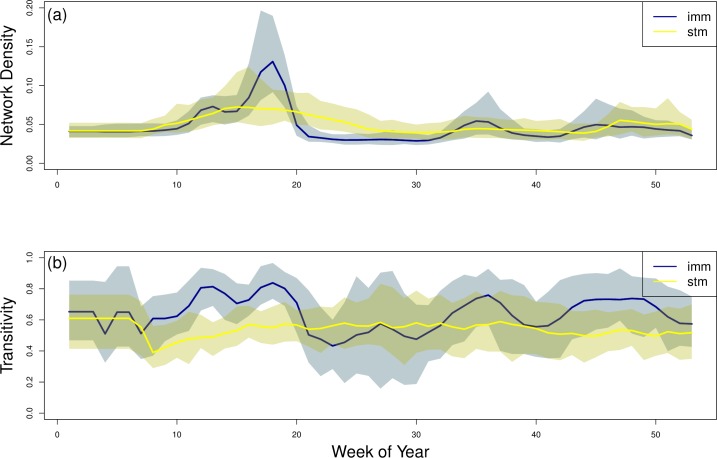
Network-wide analysis metrics of IMM and STM simulations with adjacency defined at 5 km. For 100 stochastic simulations of the IMM and 100 stochastic simulations of the STM, we calculated (a) network density and (b) transitivity. The dark line represents the average values across all simulations. The shaded areas represent the range between the the largest mean value and smallest mean value across all simulations. The metrics varied through time and varied by movement model simulated.

In order to quantify connectivity with network-level metrics, we found the density and transitivity in the weekly networks for 100 simulations of the IMM and 100 simulations of the STM (Fig 3). Density in the IMM and STM were similar, especially at the start and end of the year (Fig 3A) and mirrored the average strength results (Fig 2A); again, density values showed peaks in the IMM simulations that were not observed in the STM simulations (Fig 3A). Transitivity values were high in networks drawn from simulations of both movement models but IMM and STM values differed through time; however, the IMM values showed more peaks (Fig 3B). Similar to the individual connectivity metrics, the network-wide connectivity differed between the two movement models.

**Fig 4:**
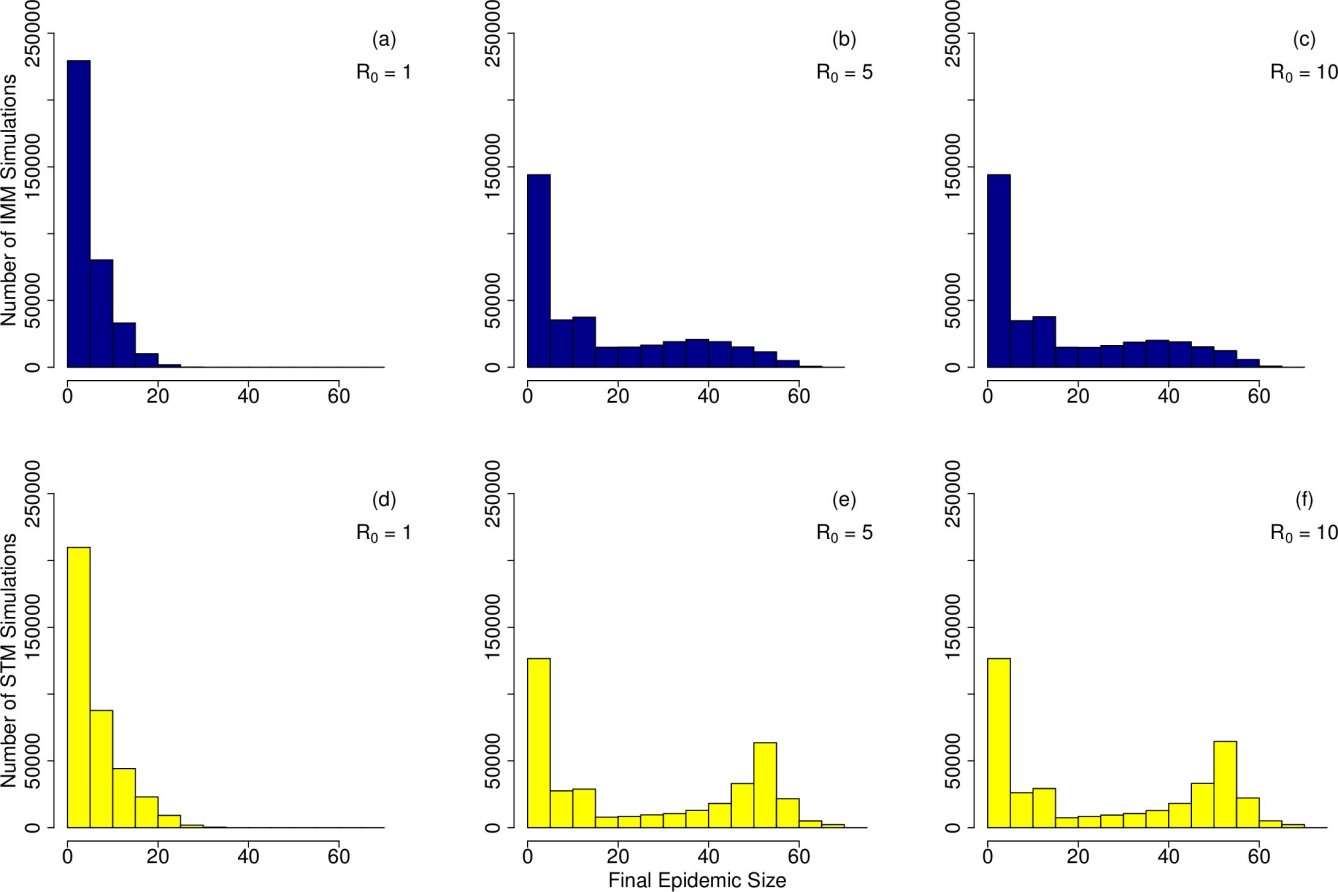
Disease simulations over IMM and STM models with adjacency defined at 5 km. We simulated disease transmission for 6 different scenarios: (a) with *R*_0_ = 1 and IMM, (b) with *R*_0_ = 5 and IMM, (c) with *R*_0_ = 10 and IMM, (d) with *R*_0_ = 1 and STM, (e) with *R*_0_ = 5 and STM, (f) with *R*_0_ = 10 and STM. Final epidemic sizes, or the total number of individuals infected over the entire course of the epidemic, are shown in the histograms. Simulations with higher values of *R*_0_ showed larger final epidemic sizes than simulations with lower values of *R*_0_ and STM simulations showed larger final epidemic sizes than IMM simulations.

In order to quantify disease transmission across all networks, we ran SIR simulations (Eqs 1 and 2) across all networks. With *R*_0_ = 1, most simulations showed small final epidemic sizes, similar to those described as stuttering chains of transmission in homogeneously mixing models, and many outbreaks failed to take off (Fig 4A and 4B). With *R*_0_ = 5, simulations showed slightly larger final epidemic sizes; still, though, many outbreaks failing to take off (Fig 4B and 4E). Results with *R*_0_ = 10 were similar to results with *R*_0_ = 5, indiciating transmission saturation of the network (Fig 4C and 4F). Transmission simulated over the networks generated from IMM output tended to produce smaller outbreaks (Fig 4A–4C) than transmission simulated over the networks generated from STM output (Fig 4D–4F) and the STM output showed a bimodal distribution at higher values of *R*_0_. Therefore, the differences in the two movement models resulted in differences in disease simulation results.

In order to correlate connectivity metrics with disease transmission, we computed the correlation coefficient for each of the five network analysis metrics and the final epidemic size produced by transmission simulations. For the IMM, we found that 3-step reach consistently and positively correlated with final epidemic size across a range of *R*_0_ values (Table 1). Betweenness centrality also showed consistent positive correlations, although the magnitude of the association was smaller (Table 1).

**Table 1:** Correlation between mean IMM network metrics with adjacency defined at 5 km and mean simulated final epidemic size.

	*R*_0_ = 1 Correlation (p-value)	*R*_0_ = 5 Correlation (p-value)	*R*_0_ = 10 Correlation (p-value)
Strength	0.054 (8.61e-5)	0.049 (3.6e-4)	0.049 (3.6e-4)
Betweenness centrality	0.092 (1.58e-11)	0.11 (1.69e-15)	0.11 (2.18e-15)
3-step reach	0.14 (< 2.2e-16)	0.16 (< 2.2e-16)	0.16 (< 2.2e-16)
Density	0.054 (8.61e-5)	0.049 (3.6e-4)	0.049 (3.6e-4)
Transitivity	0.075 (5.43e-08)	0.03 (0.038)	0.028 (0.042)

For the STM, we found a different pattern in associations. In this case, strength and density consistently and positively correlated with final epidemic size across a range of *R*_0_ values (Table 2). Transitivity also showed consistent positive correlations, although the magnitude of the association was smaller (Table 2). In this way, multiple metrics appear to correlate with disease simulation when averaged across nodes and across simulations. However, this averaging diminishes the advantages of network analyses, which excel in ability to capture individual behavior.

**Table 2:** Correlation between mean STM network metrics with adjacency defined at 5 km and mean simulated final epidemic size.

	*R*_0_ = 1 Correlation (p-value)	*R*_0_ = 5 Correlation (p-value)	*R*_0_ = 10 Correlation (p-value)
Strength	0.21 (< 2.2e-16)	0.23 (< 2.2e-16)	0.23 (< 2.2e-16)
Betweenness centrality	-0.044 (0.0012)	-0.068 (6.39e-7)	-0.069 (5.24e-07)
3-step reach	0.052 (0.00017)	0.065 (2.3e-6)	0.065 (3.0e-6)
Density	0.21 (< 2.2e-16)	0.23 (< 2.2e-16)	0.23 (< 2.2e-16)
Transitivity	0.10 (1.2e-13)	0.12 (<2.2e-16)	0.12 (<2.2e-16)

To capture temporal variation in the movement model network metrics and disease simulation results, we correlated weekly connectivity metrics with weekly outbreak results between 0 and 5 weeks lagged. We found, in general, equal or higher correlation when considering time-variation. We found that lagged strength, lagged betweenness centrality, and lagged 3-step reach correlated with final outbreak size for the IMM; lagged strength and lagged 3-step reach correlated with final outbreak size for the STM (Table 3).

**Table 3:** Correlation between time-varying network metrics with adjacency defined at 5 km and time-varying simulated final epidemic size with *R*_0_ = 5.

	IMM		STM	
	Correlation coefficient	Time lag with greatest correlation	Correlation coefficient	Time lag with greatest correlation
Strength	0.0435		0.22	
Lagged Strength	0.20	4 weeks	0.30	4 weeks
Betweenness centrality	0.10		-0.10	
Lagged betweenness centrality	0.16	1 week	-0.07	no lag
3-step reach	0.16		0.05	
Lagged 3-step reach	0.23	3 weeks	0.27	5 weeks
Density	0.18		0.13	
Lagged density	0.12	no lag	0.12	no lag
Transitivity	0.13		-0.053	
Lagged transitivity	0.11	no lag	0.063	5 weeks

## Discussion

Transmission models are often matched with models of host movement seeking greater accuracy in representing heterogeneity and other processes that drive disease spread [4–9]. Which host movement model is chosen to couple with a disease transmission model can affect incidence results. We found that network analysis metrics–strength, 3-step reach, network density–correlated with simulated transmission when network and disease dynamics were averaged across time. When network metrics show a large degree of temporal variation, lagged metrics provide even stronger correlations. However, when considering time-variation in both the movement model and disease transmission, betweenness centrality did not provide a correlation with final epidemic size. For these reasons, we can recommend mean strength, mean 3-step reach, and network density as analysis tools that can evaluate the results of a movement model and correlate with disease transmission in both time invariant and time-varying scenarios.

The practical significance of betweenness centrality measures for networks has been noted for medicine, veterinary health, and public health. Because this metric quantifies the number of shortest paths on which a node lies [35], it can be used to identify individuals to target for vaccination that would most effectively cease outbreak transmission [36], especially in assortative networks and at the start of an outbreak [37]. These works suggest that betweenness centrality is a metric that captures the characteristic of network structure that affects disease spread. It is surprising, then, that in our study, betweenness centrality very weakly correlated only when averaged across the IMM network or with time-lagged values from the IMM simulations; there were no positive correlations with any of the STM simulations. This suggests that nodes that have high betweenness centrality might change over the course of the simulations in the STM simulations. High betweenness centrality might not be an intrinsic quality of a node in this system, but it might be a product of movements, seasonality, and network structure.

Quantifying network density leads to a different set of practical applications. Interestingly, we found that this metric was time-varying. Based on the networks drawn from empirical data, we found that network density was low in August and September at the end of the rainy season. When herds begin to move to their dry season pastures, in October 2007, herds cluster together and display the highest network density of the entire year, because network density steadily declines until the following August. Because herds are geographically clustered, this mustering before dispersal presents a time period in which information or vaccinations could be efficiently disseminated among herders enrolled in this study. We quantify this seasonality in contacts and simulate its effects on transmission of an infectious disease in another study [38].

Uncertainty is inherent in location data. To address uncertainty in location data, we considered herds to be adjacent at three different distances. Adjacency defined at 5 km or 10 km can be thought of as analogous to a 5 km or 10 km error in location data. Interestingly, we found similar quantities of infected herds from disease transmission models over networks with adjacency defined at 5 km and 10 km. This suggests that precise locations of each camp or each animal are not needed to make accurate conclusions about network characteristics and disease transmission, similar to what has previous findings that full location data are not needed to determine optimal control [39]. Disease dynamics can be inferred with reasonable accuracy with datasets at less resolution than perfect observation of all animals.

Our transmission model has been formulated to represent generic pathogens that spread either by direct contact or by aerosol through a population using a simulation procedure that keeps *R*_0_ near the value set in simulations. Simulations parametrized as *R*_0_ = 1 represent the threshold value between epidemics occurring and not occurring; with the stochasticity inherent in our transmission algorithm, this results in some stuttering chains of transmission and other instances where the epidemic fails to take off. Because of the way transmission was propogated, the variation in simulation outcomes was relatively low and the number of stochastic fadeouts might have been lower than for other more random simulation procedures. For the other values of *R*_0_ simulated, these models would be especially applicable to livestock pathogens such as foot-and-mouth disease virus. Nevertheless, livestock diseases are spread by many other modes, including environmental or vector borne transmission. Conceptually, our model with *R*_0_ = 5 that assumes direct transmission will correspond to a model with a lower *R*_0_ that assumes environmental transmission, because our networks are defined by sharing geographic locations. It remains a limitation of our model that not all modes of transmission are represented and that these mobile camps might interact with livestock reared in sedentary farms or traded at markets that are not included in the system.

Nevertheless, we have found that the choice of a movement model that is coupled with a disease transmission model can influence connectivity of the host population and disease transmission results. We have found that strength, network density, and 3-step reach can be used to analyze the results of a movement model simulation in order to correlate with disease transmission simulations. We advise quantitative analysis of the movement model, evaluating sensitivity to movement and location parameters, as a requisite to multilevel modeling of disease dynamics in a mobile host population.

Overall, these results have two major implications for pathogen spread among mobile camps. First, they indicate that identity of centrally connected camps can change over time. This suggest that connectivity might not be an innate property of a camp but a property that emerges though some combination of movement, seasonality, and other factors. Second, they indicate that multiple movement models should be considered and vetted before using to make a single movement model in combination with an infectious disease transmission model for decisions about the management infectious disease transmission but exact camp locations are not needed. For this reason, models of infectious disease transmission could greatly inform preventative policies and decisions about infectious disease control.

## Supporting information

S1 Fig Individual-level network analysis metrics of IMM and STM simulations with adjacency defined at 0 km. For 100 stochastic simulations of the IMM and 100 stochastic simulations of the STM, we calculated (a) average strength, (b) average betweenness centrality, and (c) average 3-step reach. We averaged these values over all nodes in a single network. The dark line represents the average values across all simulations. The shaded areas represent the range between the maximum and minimum values across all simulations. The metrics varied through time and varied by hidden movement model simulated.
(TIF)

S2 Fig Individual-level network analysis metrics of IMM and STM simulations with adjacency defined at 10 km. For 100 stochastic simulations of the IMM and 100 stochastic simulations of the STM, we calculated (a) average strength, (b) average betweenness centrality, and (c) average 3-step reach. We averaged these values over all nodes in a single network. The dark line represents the average values across all simulations. The shaded areas represent the range between the maximum and minimum values across all simulations. The metrics varied through time and varied by hidden movement model simulated.
(TIF)

S3 Fig Network-wide metrics of IMM and STM simulations with adjacency defined at 0 km. For 100 stochastic simulations of the IMM and 100 stochastic simulations of the STM, we calculated the network density. The dark line represents the average values across all simulations. The shaded areas represent the range between the maximum and minimum values across all simulations. The metric varied through time and varied by hidden movement model simulated.
(TIF)

S4 Fig Network-wide metrics of IMM and STM simulations with adjacency defined at 10 km. For 100 stochastic simulations of the IMM and 100 stochastic simulations of the STM, we calculated (a) network density and (b) transitivity. The dark line represents the average values across all simulations. The shaded areas represent the range between the maximum and minimum values across all simulations. The metrics varied through time and varied by hidden movement model simulated.
(TIF)

S5 Fig Disease simulations over IMM and STM models with adjacency defined at 0 km. We simulated disease transmission for 6 different scenarios: (a) with *R*_0_ = 1 and IMM, (b) with *R*_0_ = 5 and IMM, (c) with *R*_0_ = 10 and IMM, (d) with *R*_0_ = 1 and STM, (e) with *R*_0_ = 5 and STM, (f) with *R*_0_ = 10 and STM. Final epidemic sizes, or the total number of individuals infected over the entire course of the epidemic, are shown in the histograms.
(TIF)

S6 Fig Disease simulations over IMM and STM models with adjacency defined at 10 km. We simulated disease transmission for 6 different scenarios: (a) with *R*_0_ = 1 and IMM, (b) with *R*_0_ = 5 and IMM, (c) with *R*_0_ = 10 and IMM, (d) with *R*_0_ = 1 and STM, (e) with *R*_0_ = 5 and STM, (f) with *R*_0_ = 10 and STM. Final epidemic sizes, or the total number of individuals infected over the entire course of the epidemic, are shown in the histograms.
(TIF)

S1 Table Correlation between mean STM network metrics with adjacency defined at 0 km and mean simulated final epidemic size. (DOCX)

S2 Table Correlation between mean IMM network metrics with adjacency defined at 10 km and mean simulated final epidemic size. (DOCX)

S3 Table Correlation between mean STM network metrics with adjacency defined at 10 km and mean simulated final epidemic size. (DOCX)
