# Supplementary figures and images for: Network analyses to quantify effects of host movement in multilevel disease transmission models using foot and mouth disease in Cameroon as a case study
Source: PLoS Comput Biol. 2019 Aug 29;15(8):e1007184. doi: 10.1371/journal.pcbi.1007184 (PMC6776348; doi:10.1371/journal.pcbi.1007184)

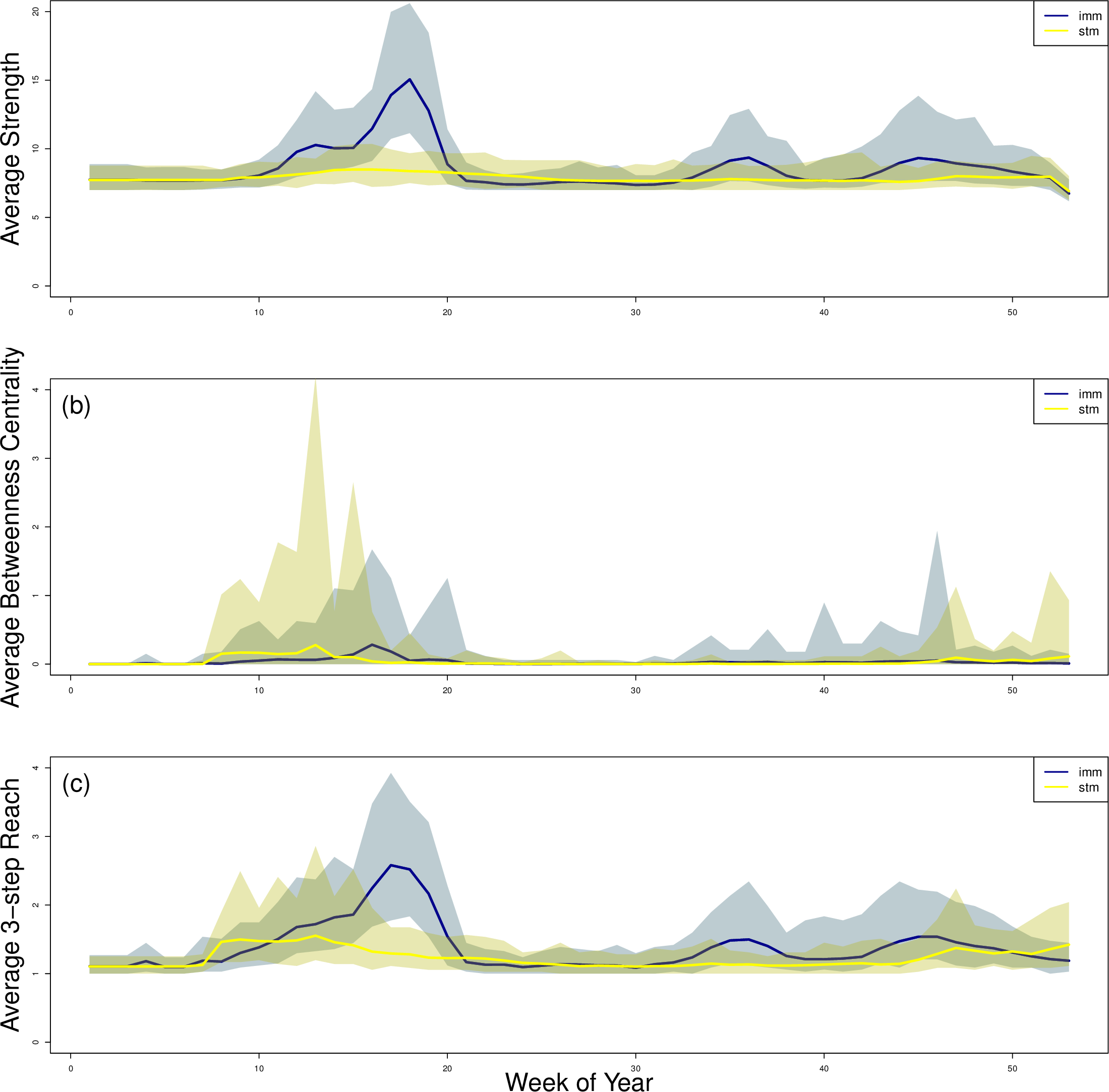

Supplement: S1 Fig — For 100 stochastic simulations of the IMM and 100 stochastic simulations of the STM, we calculated (a) average strength, (b) average betweenness centrality, and (c) average 3-step reach. We averaged these values over all nodes in a single network. The dark line represents the average values across all simulations. The shaded areas represent the range between the maximum and minimum values across all simulations. The metrics varied through time and varied by hidden movement model simulated. (TIF) [file pcbi.1007184.s001.tif]

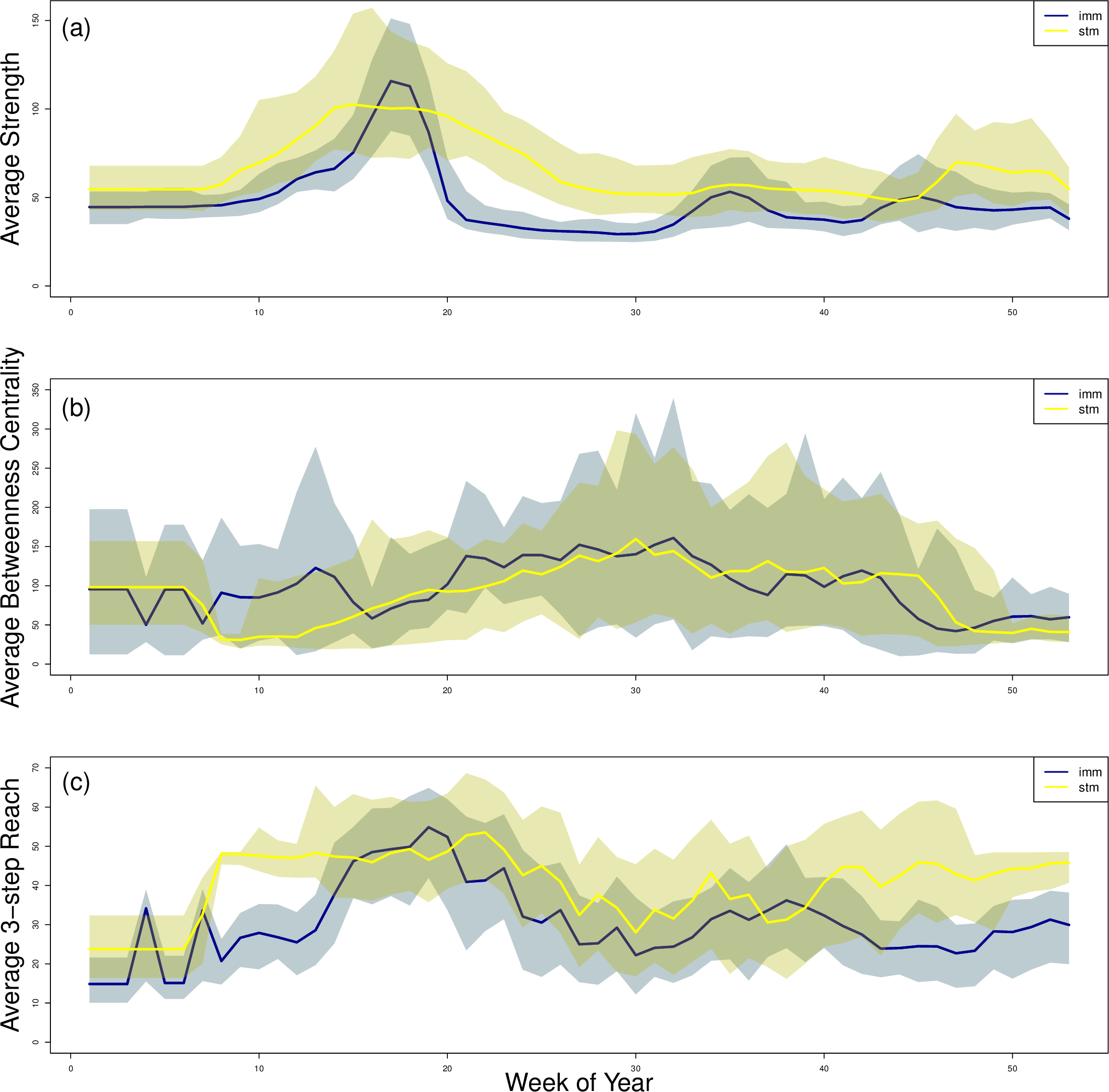

Supplement: S2 Fig — For 100 stochastic simulations of the IMM and 100 stochastic simulations of the STM, we calculated (a) average strength, (b) average betweenness centrality, and (c) average 3-step reach. We averaged these values over all nodes in a single network. The dark line represents the average values across all simulations. The shaded areas represent the range between the maximum and minimum values across all simulations. The metrics varied through time and varied by hidden movement model simulated. (TIF) [file pcbi.1007184.s002.tif]

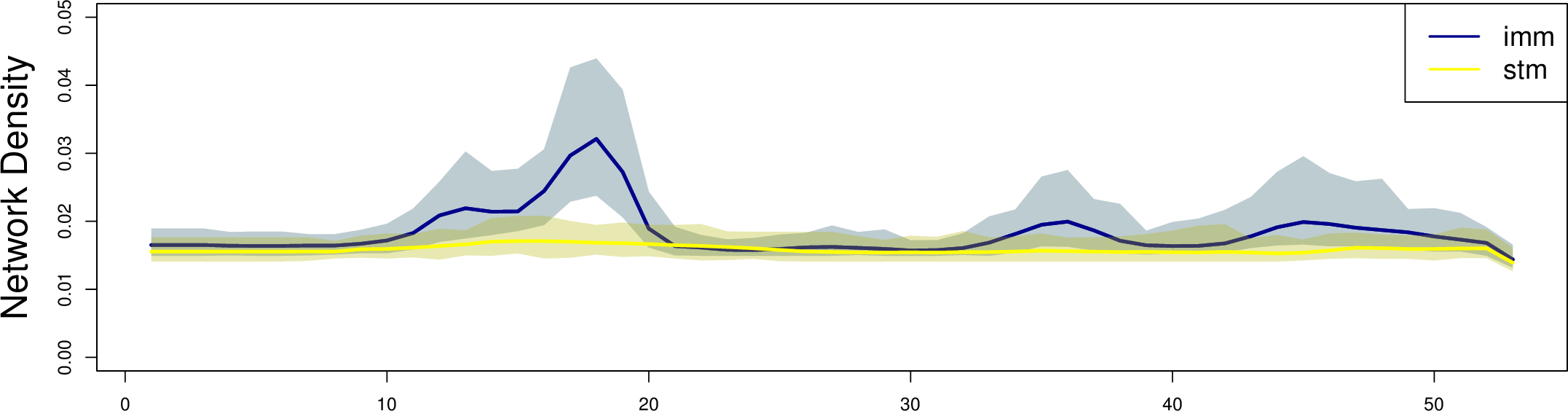

Supplement: S3 Fig — For 100 stochastic simulations of the IMM and 100 stochastic simulations of the STM, we calculated the network density. The dark line represents the average values across all simulations. The shaded areas represent the range between the maximum and minimum values across all simulations. The metric varied through time and varied by hidden movement model simulated. (TIF) [file pcbi.1007184.s003.tif]

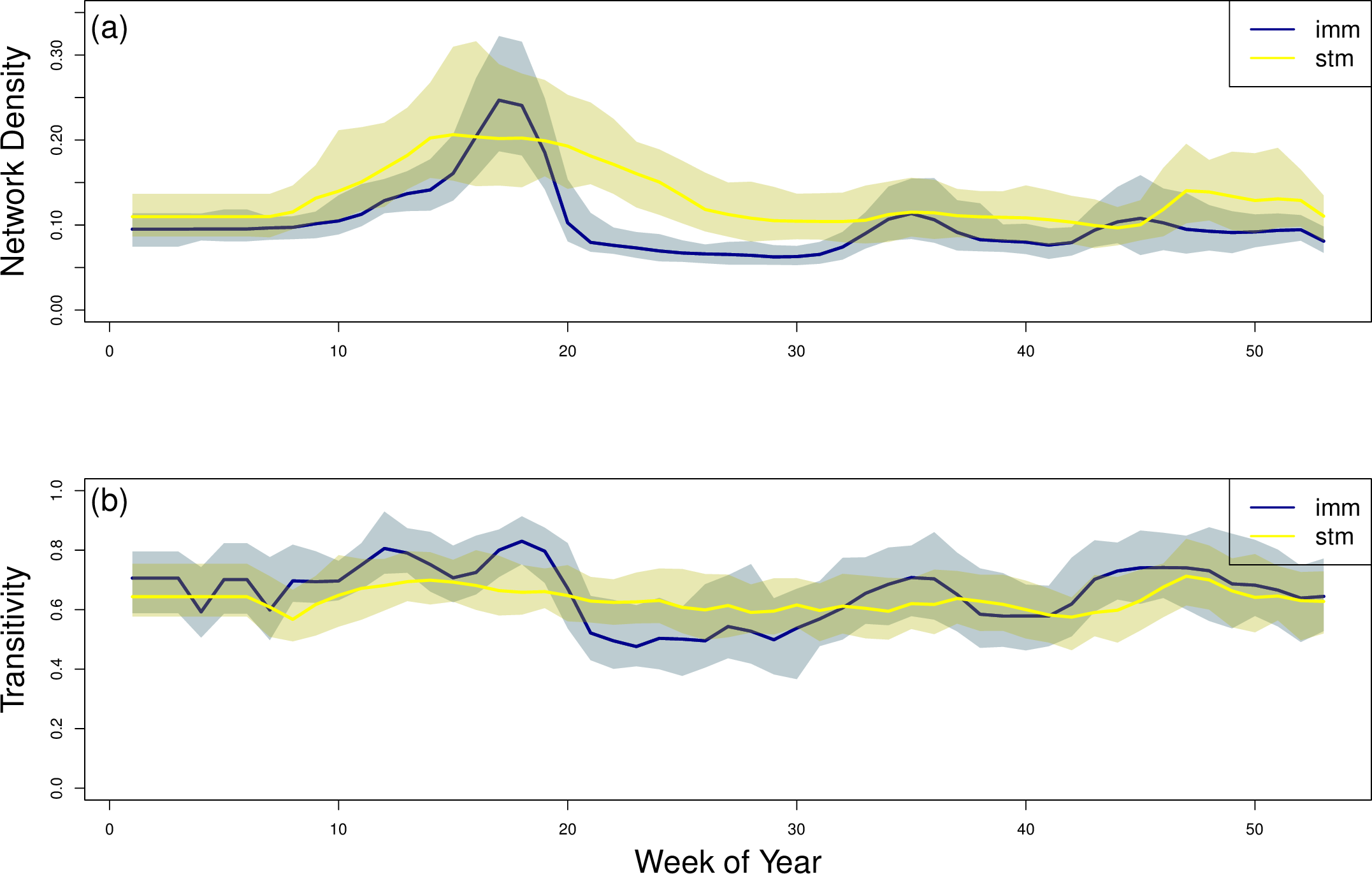

Supplement: S4 Fig — For 100 stochastic simulations of the IMM and 100 stochastic simulations of the STM, we calculated (a) network density and (b) transitivity. The dark line represents the average values across all simulations. The shaded areas represent the range between the maximum and minimum values across all simulations. The metrics varied through time and varied by hidden movement model simulated. (TIF) [file pcbi.1007184.s004.tif]

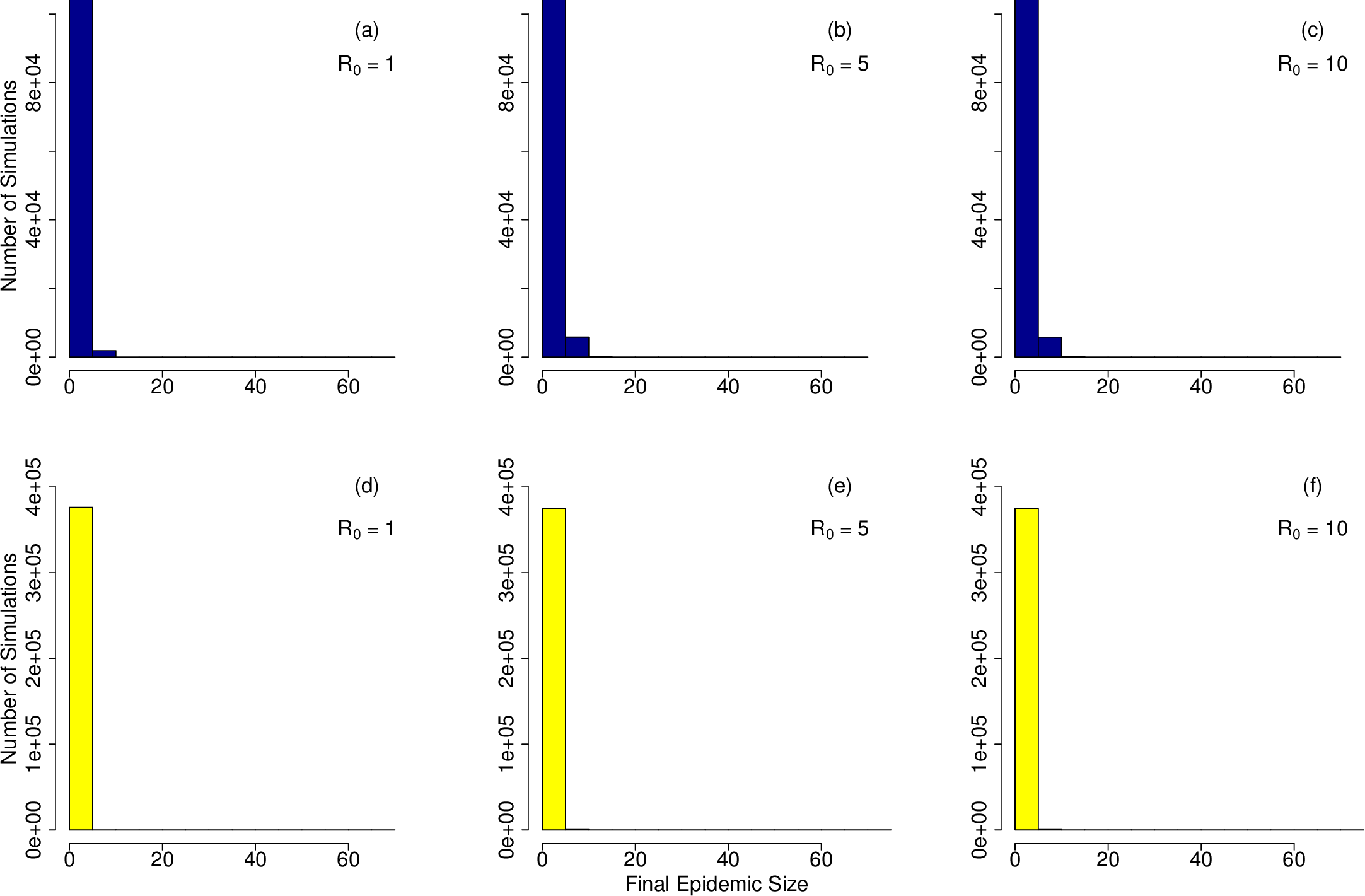

Supplement: S5 Fig — We simulated disease transmission for 6 different scenarios: (a) with R0 = 1 and IMM, (b) with R0 = 5 and IMM, (c) with R0 = 10 and IMM, (d) with R0 = 1 and STM, (e) with R0 = 5 and STM, (f) with R0 = 10 and STM. Final epidemic sizes, or the total number of individuals infected over the entire course of the epidemic, are shown in the histograms. (TIF) [file pcbi.1007184.s005.tif]

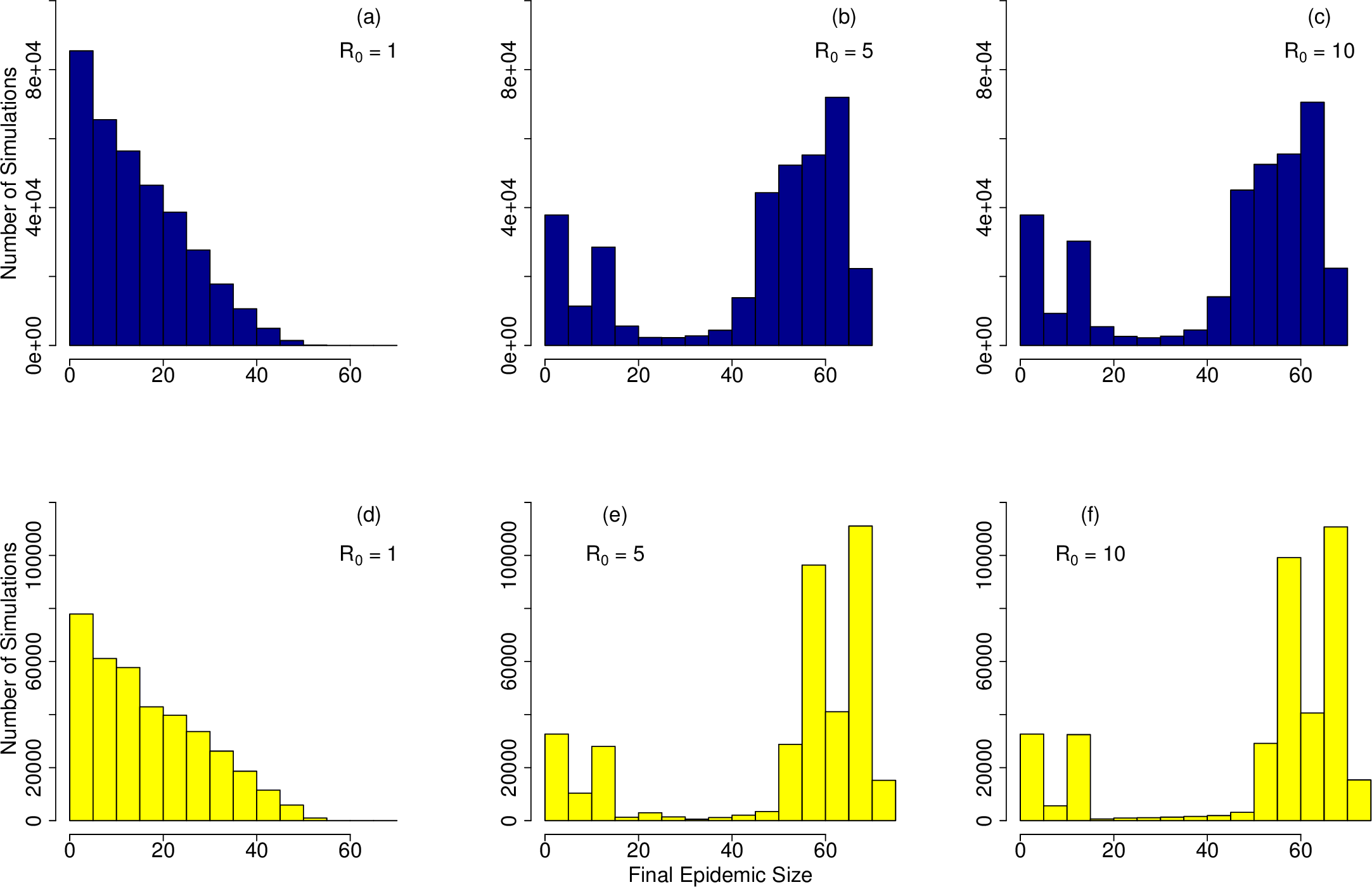

Supplement: S6 Fig — We simulated disease transmission for 6 different scenarios: (a) with R0 = 1 and IMM, (b) with R0 = 5 and IMM, (c) with R0 = 10 and IMM, (d) with R0 = 1 and STM, (e) with R0 = 5 and STM, (f) with R0 = 10 and STM. Final epidemic sizes, or the total number of individuals infected over the entire course of the epidemic, are shown in the histograms. (TIF) [file pcbi.1007184.s006.tif]
