# Supplementary material for: Network analyses to quantify effects of host movement in multilevel disease transmission models using foot and mouth disease in Cameroon as a case study
Source: PLoS Comput Biol. 2019 Aug 29;15(8):e1007184. doi: 10.1371/journal.pcbi.1007184 (PMC6776348; doi:10.1371/journal.pcbi.1007184)
Supplement: S1 Table — (DOCX) [file pcbi.1007184.s007.docx]

**Table S1. Correlation between mean STM network metrics with adjacency defined at 0 km and mean simulated final epidemic size**

|  | $\boldsymbol{R}_{\boldsymbol{0}}\boldsymbol{=1}$  Correlation (p-value) | $\boldsymbol{R}_{\boldsymbol{0}}\boldsymbol{=5}$  Correlation (p-value) | $\boldsymbol{R}_{\boldsymbol{0}}\boldsymbol{=10}$  Correlation (p-value) |
| --- | --- | --- | --- |
| Strength | 0.10 (7.07e-14) | 0.11 (1.34e-14) | 0.11 (1.29e-14) |
| Betweenness centrality | 0.011 (0.44) | 0.031 (0.023) | 0.031 (0.022) |
| 3-step reach | 0.021 (0.12) | 0.029 (0.037) | 0.029 (0.036) |
| Density | 0.10 (7.07e-14) | 0.11 (1.34e-14) | 0.11 (1.29e-14) |
