# Supplementary material for: Network analyses to quantify effects of host movement in multilevel disease transmission models using foot and mouth disease in Cameroon as a case study
Source: PLoS Comput Biol. 2019 Aug 29;15(8):e1007184. doi: 10.1371/journal.pcbi.1007184 (PMC6776348; doi:10.1371/journal.pcbi.1007184)
Supplement: S2 Table — (DOCX) [file pcbi.1007184.s008.docx]

**Table S2. Correlation between mean IMM network metrics with adjacency defined at 10 km and mean simulated final epidemic size**

|  | $\boldsymbol{R}_{\boldsymbol{0}}\boldsymbol{=1}$  Correlation (p-value) | $\boldsymbol{R}_{\boldsymbol{0}}\boldsymbol{=5}$  Correlation (p-value) | $\boldsymbol{R}_{\boldsymbol{0}}\boldsymbol{=10}$  Correlation (p-value) |
| --- | --- | --- | --- |
| Strength | 0.062 (5.759e-06) | 0.031 (0.024) | 0.028 (0.038) |
| Betweenness centrality | -0.067 (9.35e-07) | -0.025 (0.073) | -0.023 (0.099) |
| 3-step reach | 0.25 (< 2.2e-16) | 0.30 (< 2.2e-16) | 0.30 (< 2.2e-16 |
| Density | 0.062 (5.759e-06) | 0.031 (0.024) | 0.028 (0.038) |
| Transitivity | -0.050 (0.00036) | -0.12 (< 2.2e-16) | -0.13 (< 2.2e-16) |
