# Supplementary material for: Network analyses to quantify effects of host movement in multilevel disease transmission models using foot and mouth disease in Cameroon as a case study
Source: PLoS Comput Biol. 2019 Aug 29;15(8):e1007184. doi: 10.1371/journal.pcbi.1007184 (PMC6776348; doi:10.1371/journal.pcbi.1007184)
Supplement: S3 Table — (DOCX) [file pcbi.1007184.s009.docx]

**Table S3. Correlation between mean STM network metrics with adjacency defined at 10 km and mean simulated final epidemic size**

|  | $\boldsymbol{R}_{\boldsymbol{0}}\boldsymbol{=1}$  Correlation (p-value) | $\boldsymbol{R}_{\boldsymbol{0}}\boldsymbol{=5}$  Correlation (p-value) | $\boldsymbol{R}_{\boldsymbol{0}}\boldsymbol{=10}$  Correlation (p-value) |
| --- | --- | --- | --- |
| Strength | 0.23 (< 2.2e-16) | 0.26 (< 2.2e-16) | 0.26 (< 2.2e-16) |
| Betweenness centrality | -0.028 (0.03826) | -0.018 (0.1871) | -0.018 (0.1951) |
| 3-step reach | -0.032 (0.01846) | -0.042 (0.002041) | -0.044 (0.001407) |
| Density | 0.23 (< 2.2e-16) | 0.26 (< 2.2e-16) | 0.26 (< 2.2e-16) |
| Transitivity | 0.056 (4.943e-05) | 0.043 (0.001776) | 0.045 (0.0009527) |
